# Smoking, mortality, access to diagnosis, and treatment of lung cancer in Brazil

**DOI:** 10.11606/s1518-8787.2024058005704

**Published:** 2024-04-25

**Authors:** Mônica Rodrigues Campos, Jessica Muzy Rodrigues, Aline Pinto Marques, Lara Vinhal Faria, Tayná Sequeira Valerio, Mario Jorge Sobreira da Silva, Debora Castanheira Pires, Luisa Arueira Chaves, Carlos Henrique Dantas Cardoso, Silvio Rodrigues Campos, Isabel Cristina Martins Emmerick

**Affiliations:** I Fundação Oswaldo Cruz Escola Nacional de Saúde Pública Sérgio Arouca Departamento de Ciências Sociais Rio de Janeiro RJ Brasil Fundação Oswaldo Cruz. Escola Nacional de Saúde Pública Sérgio Arouca. Departamento de Ciências Sociais. Rio de Janeiro, RJ, Brasil; II Fundação Oswaldo Cruz Instituto de Comunicação e Informação Científica e Tecnológica em Saúde Laboratório de Informação em Saúde Rio de Janeiro RJ Brasil Fundação Oswaldo Cruz. Instituto de Comunicação e Informação Científica e Tecnológica em Saúde. Laboratório de Informação em Saúde. Rio de Janeiro, RJ, Brasil; III Fundação Oswaldo Cruz Escola Nacional de Saúde Pública Sérgio Arouca Programa de Pós-Graduação em Saúde Pública Rio de Janeiro RJ Brasil Fundação Oswaldo Cruz. Escola Nacional de Saúde Pública Sérgio Arouca. Programa de Pós-Graduação em Saúde Pública. Rio de Janeiro, RJ, Brasil; IV Instituto Nacional de Câncer Divisão de Ensino Rio de Janeiro RJ Brasil Instituto Nacional de Câncer. Divisão de Ensino. Rio de Janeiro, RJ, Brasil; V Fundação Oswaldo Cruz Instituto Nacional de Infectologia Evandro Chagas Laboratório de Pesquisa Clínica em DST e Aids Rio de Janeiro RJ Brasil Fundação Oswaldo Cruz. Instituto Nacional de Infectologia Evandro Chagas. Laboratório de Pesquisa Clínica em DST e Aids. Rio de Janeiro, RJ, Brasil; VI Universidade Federal do Rio de Janeiro Instituto de Ciências Farmacêuticas Macaé RJ Brasil Universidade Federal do Rio de Janeiro. Instituto de Ciências Farmacêuticas. Macaé, RJ, Brasil; VII Universidade Federal do Rio de Janeiro Instituto de Educação em Ciências e Saúde Programa de Pós-Graduação em Educação, Ciências e Saúde Rio de Janeiro RJ Brasil Universidade Federal do Rio de Janeiro. Instituto de Educação em Ciências e Saúde. Programa de Pós-Graduação em Educação, Ciências e Saúde. Rio de Janeiro, RJ, Brasil; VIII Fundação Oswaldo Cruz Escola Nacional de Saúde Pública Sérgio Arouca Rio de Janeiro RJ Brasil Fundação Oswaldo Cruz. Escola Nacional de Saúde Pública Sérgio Arouca. Rio de Janeiro, RJ, Brasil; IX University of Massachusetts UMass Chan Medical School Department of Surgery Worcester MA Estados Unidos University of Massachusetts. UMass Chan Medical School. Department of Surgery. Worcester, MA, Estados Unidos

**Keywords:** Tobacco Use Disorder, Lung Neoplasms, Health Information Systems, Mortality Registries

## Abstract

**INTRODUCTION:**

Lung cancer (LC) is a relevant public health problem in Brazil and worldwide, given its high incidence and mortality. Thus, the objective of this study is to analyze the distribution of smoking and smoking status according to sociodemographic characteristics and disparities in access, treatment, and mortality due to LC in Brazil in 2013 and 2019.

**METHOD:**

Retrospective study of triangulation of national data sources: a) analysis of the distribution of smoking, based on the National Survey of Health (PNS); b) investigation of LC records via Hospital-based Cancer Registry (HCR); and c) distribution of mortality due to LC in the Mortality Information System (SIM).

**RESULTS:**

There was a decrease in the percentage of people who had never smoked from 2013 (68.5%) to 2019 (60.2%) and in smoking history (pack-years). This was observed to be greater in men, people of older age groups, and those with less education. Concerning patients registered in the HCR, entry into the healthcare service occurs at the age of 50, and only 19% have never smoked. While smokers in the population are mainly Mixed-race, patients in the HCR are primarily White. As for the initial stage (I and II), it is more common in White people and people who have never smoked. The mortality rate varied from 1.00 for people with higher education to 3.36 for people without education. Furthermore, White people have a mortality rate three times higher than that of Black and mixed-race people.

**CONCLUSION:**

This article highlighted relevant sociodemographic disparities in access to LC diagnosis, treatment, and mortality. Therefore, the recommendation is to strengthen the Population-Based Cancer Registry and develop and implement a nationwide LC screening strategy in Brazil since combined prevention and early diagnosis strategies work better in controlling mortality from the disease and continued investment in tobacco prevention and control policies.

## INTRODUCTION

Lung cancer (LC) has been the most common malignancy worldwide since 1985, both in incidence and mortality^1^. In 2020, it was estimated that around 12% of new cancer cases were attributed to LC, which was responsible for 18.4% of cancer deaths worldwide^1^. Furthermore, there is high lethality and low survival after diagnosis, especially at an advanced stage^2^.

In Brazil, estimates for the period 2023-2025 indicate that LC will be the second most common type of cancer, not counting specific breast and prostate malignancies^3^. In 2020, it was also responsible for the leading cause of cancer-related mortality ^4^.

The main risk factor for the development of LC is smoking^5^, often associated with age, due to the length of exposure to tobacco^4^. Other related factors are occupational risk^6^, environmental exposure^7^, and sociodemographic characteristics, such as sex^8^, race^9^, and education^10^. Notably, in Brazil, there is a gap in the literature regarding factors other than smoking.

Understanding how these factors affect the risk of developing LC, their association with tobacco consumption in Brazil, and the possible impact on mortality is essential for adequately formulating public policies. A promising way to reduce LC disparities is to improve prevention and detection of the disease at an early stage^11^, mainly through screening programs for people in at-risk groups^12^. The criteria for establishing an adequate screening program depend on understanding the degree of vulnerability of a population and the disparities in their access to diagnosis and treatment^12^.

Age and smoking status are generally the main criteria for defining high-risk groups in LC screening guidelines. However, these parameters must be adjusted considering the demographic composition, specificities, and other relevant markers such as socioeconomic situation^12^.

For instance, the Chinese guidelines were adapted concerning age and smoking status (currently 50 to 74 years old, with 30 pack-years) and consider the effects of air pollution due to environmental exposure and genetic factors^13^. In the USA, the United States Preventive Services Task Force (USPSTF) guidelines were updated between 2013 and 2021 to mitigate access problems for black people. The age range was expanded to 50 to 80 years (previously 55 to 80), and the smoking burden was reduced from 30 to 20 pack-years^14^.

A valuable method for understanding the population at risk is triangulation through secondary databases, which, in addition to being easily accessible and with national coverage, allow for sociodemographic characterization and the assessment of health aspects and lifestyle habits. The Mortality Information System (SIM) is systematically used in Brazil to study LC^4,15,16^. However, two significant challenges remain: estimating the risk of developing LC and measuring its disparities in diagnosis and treatment.

The most critical item for risk estimation is calculating the smoking burden, which is possible using data from the National Survey of Health (PNS)^17^. Although the *Vigilância de Fatores de Risco e Proteção para Doenças Crônicas por Inquérito Telefônico* (VIGITEL – Surveillance of Risk and Protective Factors for Chronic Diseases by Telephone Survey) provides specific information about chronic diseases and their risk factors, its application is only carried out in capital cities, making it difficult to extrapolate results at a national level^18^. On the other hand, disparities in diagnosis and treatment can be mapped using the Hospital-Based Cancer Registry (HCR), which enables analysis according to sociodemographic, epidemiological, and clinical characteristics^19^.

This article aims to analyze the population distribution of smoking, smoking burden, LC cases and mortality, and disparities in its access and treatment, according to sociodemographic characteristics in Brazil from 2013 to 2019, to support adequate screening of LC in the country.

## METHODS

A retrospective study triangulated the PNS, HCR, and SIM as national secondary data sources. The HCR is a national database for systematic and continuous information collection from patients treated in hospital units with a confirmed cancer diagnosis. Sending data is mandatory for hospitals qualified in Specialized Oncology Care of the Brazilian Unified Health System (SUS) and optional for those not qualified^20^.

Base triangulation was carried out following the following steps:

- population analysis of the distribution of smoking (smokers, ex-smokers, smoking history, and never smoked) – PNS (2013 and 2019).
- investigation of diagnosed LC cases according to smoking status and staging (initial and advanced) – HCR^20^ (2013 and 2019).
- distribution of deaths and the mortality rate of LC – SIM (2013 to 2019).

The following were carried out to improve the quality of the data used: redistribution procedures for missing smoking and race/color data by federative unit (FU) in the HCR; correction of missing data in the staging variable (HCR) by redistribution, according to sex, age group, smoking status, and first treatment; and, in SIM, correction of garbage codes for LC, correction of ill-defined causes, correction of under-registration and redistribution of missing data by sex, age and UF. The International Classification of Disease (ICD) garbage codes refer to undefined or incomplete diagnoses that do not accurately indicate the cause of death or hospitalization^21^.

All analyses were described according to demographic and socioeconomic variables: sex, age group, race/color, and education. Sex included the categories “male” or “female” and the age group classified as a) up to 49 years old, b) 50 to 59 years old, c) 60 to 69 years old, d) 70 to 79 years old; and e) 80 years or over. For “race/color,” only the categories a) White, b) Black, or c) Mixed race were used since the contingent of Yellow and Indigenous people in the PNS was very small, both in 2013 (1.6%) and in 2019 (2.5%). The education variable was recategorized as a) no education, b) incomplete primary, c) complete primary or incomplete secondary, d) complete secondary, e) incomplete higher education, and f) complete higher education. Calculating the LC mortality rate by race and education required estimating the Brazilian population for 2013 and 2019, applying the percentage distribution of the education and race/color variables from the PNS in the preliminary population estimates prepared by the Ministry of Health, *Secretaria de Vigilância em Saúde* (SVS – Health Surveillance Secretariat), *Departamento de Análise Epidemiológica e Vigilância de Doenças Não Transmissíveis* (DAENT – Department of Epidemiological Analysis and Surveillance of Noncommunicable Diseases), and *Coordenação-Geral de Informações e Análises Epidemiológicas* (CGIAE – General Coordination of Epidemiological Information and Analysis).

The Statistical Package for Social Science for Windows (SPSS) version 21 was used for the PNS analyses, the SAS studio and phyton were used for HCR, and the SPSS version 21 and phyton were used for SIM.

### Methodological aspects of estimating smoking and smoking status in the PNS

Adult residents (≥ 18 years old) who responded to the specific questionnaire about their health status, lifestyle, and chronic diseases were selected, totaling 60,202 respondents in 2013 and 88,943 in 2019. The reweighted version of the PNS-2013 was used, aiming for comparison with 2019.

Regarding the definition/elaboration of the variables arising from the PNS, the following question was used to identify smokers: “Do you currently smoke any tobacco products?” Those who answered “Yes, daily” or “Yes, less than daily” were considered smokers. Former smokers were identified based on the question: “And in the past, did you smoke any tobacco products?” considering the answers “Yes, daily” or “Yes, less than daily.” Non-smokers were those who answered “No, I have never smoked” to the previous question.

The smoking burden was estimated for people who smoked daily and who consumed “only industrialized cigarettes,” “only straw or hand-rolled cigarettes,” or both. Sporadic smokers and ex-smokers did not answer questions about the age at which they started smoking and the amount consumed. Approximately 10% of ex-smokers (n = 2,102) reported having quit smoking before establishing a habit, being disregarded in the calculation of smoking burden.

The smoking burden was estimated in pack-years. This number is a synthetic measure that combines the duration and intensity of smoking: the smoking burden of a pack-year corresponds to the daily smoking of 20 cigarettes for a year^22^. The conversion was carried out to equate industrialized cigarettes with straw or hand-rolled cigarettes, whose consumption was multiplied by three. Despite the lack of robust literature on the equivalence between them, experts point out that one straw cigarette is equivalent to three industrial cigarettes^23,24^. The result was classified into the categories: a) up to 19 pack-years, b) from 20 to 29 pack-years, and c) 30 pack-years or more.

### Methodological aspects of SIM corrections

Missing data on sex and age was imputed. The most frequent response category in the database (male – 810 cases) was used to impute sex gaps. Regarding age, out of 215,247 LC deaths in individuals over 18 years (2013 – 2019), 4,719 unfilled cases were imputed using the median age of valid cases.

The garbage codes were corrected, checking the codes of interest LC (ICD C34) and their percentage of redistribution proposed by Malta et al.^16^. The distribution of garbage codes was recorded to assess their absolute frequency in each subgroup, according to the macro-region of residence and age, to be used in the redistribution destination. After this survey, n = 4,719 garbage code-type deaths were redistributed.

Deaths coded as ill-defined causes refer to chapter “R” of ICD-10 (R00-R99). The redistribution was carried out according to the proportional distribution of causes, verified among the ill-defined causes investigated and reclassified, according to the coefficient proposed by França et al.^15^, according to region, age group, and sex. After investigation for ICD-C34, 12,992 ill-defined causes were reassigned.

The last correction step in SIM was implementing the correction factor for under-registration of deaths, according to the methodology proposed by the *Rede Interagencial de Informações para a Saúde* (RIPSA – Interagency Health Information Network)^25^. The total quantified after correction was 10,176 LC deaths compared with those reported initially (n = 203,986).

After carrying out the procedures mentioned above, the SIM correction percentage was 0.03% for the sex variable, 2% for age, 2% for garbage codes, 6% for ill-defined causes, and 4.7% for sub-registration.

## RESULTS

Table 1 presents the percentage distribution of smokers, ex-smokers, people who have never smoked, and smoking burden according to sociodemographic variables in 2013 and 2019. There was a reduction in the percentage of people who never smoked from (68.5% (2013) to 60.2% (2019) and an increase in ex-smokers from 17% (2013) to 27.1% (2019). The percentage of smokers showed a slight reduction between years, from 14.5% (2013) to 12.7% (2019).

**Table 1:** Percentage distribution of the population by sociodemographic characteristics, according to smoking status and smoking burden in the National Survey of Health (PNS). Brazil, 2013 and 2019.

Characteristic	Proportion of smokers				Smoking burden		
	
	Smokers	Ex-smokers	Never smoked	Total	Up to 19 pack-years	20 pack-years or more	Total
2013							
Total (n)	8,729	10,258	41,215	60,202	9,699	5,324	15,023
Total (% row)	14.5	17	68.5	100	64.6	35.4	100
Sex							
Male	57.2	52.6	37.7	43.1	50.5	63.7	55.2
Female	42.8	47.4	62.3	56.9	49.5	36.3	44.8
Age group							
49 years or younger	62.1	44.6	72.8	66.4	58.5	32.9	49.4
50 to 59 years old	21.8	22.4	11.7	15	19.2	31.1	23.4
60 to 69 years old	10.9	18.6	8.2	10.4	12.8	22.3	16.1
70 to 79 years old	4.2	10.5	4.8	5.7	6.8	10.8	8.2
80 years or older	1.1	3.9	2.4	2.5	2.7	3	2.8
Color or race							
White	35.5	39.9	41	40	37.7	41.1	38.9
Black	11.4	9.1	9	9.4	10.3	10.2	10.3
Mixed	51.3	49.2	48.5	49	50.2	47.3	49.2
Yellow or Indigenous	1.7	1.8	1.5	1.6	1.8	1.4	1.7
Education							
No education	14.2	14.7	7.4	9.6	13.5	18.4	15.2
Incomplete primary	41.1	39.8	25.7	30.4	39	46.3	41.6
Complete primary or incomplete secondary	16	13.3	15.7	15.3	15.9	11.6	14.4
Complete secondary	17.9	18.8	30.6	26.8	18.9	14	17.1
Incomplete higher education	3.4	3.1	5.9	5	3.5	2	2.9
Complete higher education	7.5	21.6	21.2	20.5	9.2	7.7	8.7
2019							
Total (n)	11,286	23,950	53,295	88,531	21,587	9,362	30,949
Total (% row)	12.7	27.1	60.2	100	69.8	30.2	100
Sex							
Male	60.3	48.8	43.4	47.1	46.9	63.5	51.9
Female	39.7	51.2	56.6	52.9	53.1	36.5	48.1
Age group (years)							
49 or younger	53.6	40.4	64.6	56.6	51.6	23.1	42.9
50 to 59	22.7	20.7	15.3	17.7	19.3	27.6	21.8
60 to 69	16	20.6	10.9	14.2	15.5	29.3	19.7
70 to 79	6.1	12.8	6.4	8.1	9.3	15.1	11.1
80 or older	1.6	5.5	2.8	3.4	4.3	4.9	4.5
Color or race							
White	32.9	36.6	37.4	36.6	34.4	39.1	35.8
Black	13.4	12.2	10.7	11.4	12.9	12.3	12.7
Mixed	52.1	49.5	50.5	50.4	51.1	47.2	49.9
Yellow or Indigenous	1.6	1.7	1.4	1.5	1.7	1.4	1.6
Education							
No education	12.8	12	6.2	8.6	11.3	15.6	12.6
Incomplete primary	41.6	37.8	26.6	31.6	36.5	47.5	39.8
Complete primary or incomplete secondary	15.4	12.7	13.6	13.6	14.2	11.5	13.4
Complete secondary	18.5	22.2	30	26.4	22.9	15.3	20.6
Incomplete higher education	3.3	3.5	5.1	4.5	4	1.7	3.3
Complete higher education	8.3	11.8	18.5	15.4	11	8.5	10.2

Source: National Survey of Health (PNS), 2013 and 2019.

There was a reduction in the smoking burden between 2013 and 2019, and women consistently smoked less than men. Male participation among smokers increased, which was already high in 2013 (57.2%), with a significant smoking burden of over 20 pack-years (63.7%). The most significant portion of those who “never smoked” were female in 2013 (62.3%) and 2019 (56.6%). The distribution of ex-smokers by sex is similar in both years.

An increase in the percentage of ex-smokers aged 60 and over was identified between 2013 and 2019. Conversely, in 2019, the smoking burden among people in this age group was higher. The internal distribution of smokers and smoking burden according to race/color is similar to the general distribution of the population in both years. Considering race/color, Black or mixed-race individuals have a lower smoking burden when compared with white counterparts, especially in 2019. The high smoking burden among people with less education in both years is also noteworthy.

Table 2 shows the percentage distribution of smokers, ex-smokers, and people who never smoked among patients registered in the HCR in 2013 and 2019. In the HCR, the percentage of people who never smoked was, on average, 19%, considerably lower than that observed in the general population (68.5% in 2013 and 60.2% in 2019). Entry into the health service starts at 50 years of age, and the percentage of people in the most advanced age group (80 years or older) is meager compared with other ages, especially among smokers (3.4% in both years).

**Table 2:** Percentage distribution of patients diagnosed with lung cancer in the Hospital-based Cancer Registry (HCR) by sociodemographic characteristics, according to smoking status. Brazil, 2013 and 2019.

Characteristic	2013				2019			
	
	Smokers	Ex-smokers	Never smoked	Total	Smokers	Ex-smokers	Never smoked	Total
Total (n)	4,623	4,413	2,147	11,183	3,727	3,910	1,886	9,523
Total (% row)	41.3	39.5	19.2	100	39.1	41.1	19.8	100
Sex								
Male	62	65.2	38.7	58.8	58.8	65.4	36.8	57.1
Female	38	34.2	61.3	41.2	41.2	34.6	63.2	42.9
Age group (years)								
49 or younger	7.8	5.3	20.2	9.2	5.6	4.1	15	6.9
50 to 59	29.2	20.5	20	24	24.8	16.9	17.3	20.1
60 to 69	31.6	32.6	24.3	30.5	36.8	36.7	27.2	34.9
70 to 79	28.1	34.5	27.4	30.5	29.4	34.5	32.5	32.1
80 or older	3.4	7.1	8.1	5.8	3.4	7.9	8	6.1
Color or race								
White	41.7	66.2	68.7	56.5	40.6	65.6	63.5	55.3
Black	6.1	6.5	4.4	5.9	5.7	6.3	4.6	5.7
Mixed	51.1	26.5	26.5	36.7	52.5	27.2	31.4	38
Yellow or Indigenous	1.1	0.8	0.4	0.9	1.2	0.9	0.4	0.9
Education								
No education	7.8	8.8	7.8	8.2	7.1	7.1	6.6	7
Incomplete primary	33.3	33.4	31.1	32.9	38.9	37.4	37	37.9
Complete primary or incomplete secondary	15.2	15.7	16.6	15.6	15.1	14.6	13.5	14.6
Complete secondary	10.6	10.8	13.5	11.3	12.8	12.7	15.7	13.3
Incomplete higher education	0.4	0.5	0.8	0.5	0.5	0.7	0.7	0.6
Complete higher education	6.2	5.8	5.9	6	4.8	5.1	6.2	5.2
No information	26.5	9.8	4.7	25.5	20.8	22.4	20.2	21.4

Source: Hospital-based Cancer Registry (HCR), 2013 and 2019.

As in the PNS, in the sex distribution of smokers and ex-smokers, men are predominant, both in 2013 (62% and 65.2%) and in 2019 (58.8% and 65.4%). Women are the majority of those who have never smoked (61.3% in 2013 and 63.2% in 2019). The difference between the distribution by race/color stands out, with a predominance of mixed-race people in the PNS (49% in 2013 and 50.4% in 2019) and most White patients in the HCR (56.5% in 2013 and 55,3% in 2019).

Despite the high percentage of brown and ex-smokers in the general population (Table 1), they are not captured in the same way in the HCR, indicating an underrepresentation. On the other hand, White ones, even ex-smokers or those who have never smoked, have more access to diagnosis and treatment, representing a more significant portion of the HCR concerning the population distribution (PNS). Regarding education, the high percentage of incomplete completion of this variable in the years (25.5% and 21.4%) draws attention to the detriment of its importance as a socioeconomic proxy for analyzing disparities in access.

Table 3 shows the level of LC staging in HCR patients in 2013 and 2019. The low percentage of people with initial staging levels (I and II) in 2013 (14.2%) and 2019 (14.5%) stands out. The distribution of sociodemographic characteristics of the population accessing services is similar between the years analyzed. In 2019, the percentage of white people with higher education and who never smoked was higher in stages I and II (58.2%, 6.8%, and 22.6%). Regarding treatment, a high percentage of first surgical treatment was observed in people with stages I and II in 2013 (45.6%) and 2019 (50.2%).

**Table 3:** Percentage distribution of patients diagnosed with lung cancer in the Hospital-based Cancer Registry (HCR) by sociodemographic characteristics, according to initial (I and II) and advanced (III and IV) staging levels. Brazil, 2013 and 2019.

Characteristic	2013				2019			
	
	No information	I and II	III and IV	Total	No information	I and II	III and IV	Total
Total (n)	1,094	1,705	8,384	11,183	832	1,587	7,104	9,523
Total (% row)	9.8	15.2	75	100	8.7	16.7	74.6	100
Sex								
Male	55.8	55.8	59.8	58.8	57.7	50.7	57.1	56.1
Female	44.2	44.2	40.2	41.2	42.3	49.3	42.9	43.9
Age group (years)								
49 or younger	10.7	9.7	8.9	9.2	7.7	6.9	6.7	6.9
50 to 59	21.8	21.6	24.8	24	17.7	17.4	20.2	19.5
60 to 69	27.4	31.3	30.8	30.6	34	34.7	34.8	34.7
70 to 79	32.2	32.4	29.9	30.5	30.8	34.4	32.3	32.5
80 or older	8	5	5.6	5.8	9.9	6.7	6	6.4
Color or race								
White	56.8	57.9	56.2	56.5	54.1	57.9	54.6	55.1
Black	4.9	6.5	5.9	5.9	5.9	4.1	5.9	5.6
Mixed	37.3	34.6	37	36.7	38.8	36.9	38.7	38.4
Yellow or Indigenous	1	1.1	0.9	0.9	1.2	1.1	0.8	0.2
Education								
No education	9.7	7.6	8.1	8.2	8.2	5.6	7.2	7
Incomplete primary	27.5	30.1	34.2	32.9	33.1	33.3	39.5	37.9
Complete primary or incomplete secondary	14.2	14.4	16.1	15.6	3	12	45	14.6
Complete secondary	9	11.7	11.5	11.3	9.1	14.9	15.1	13.3
Incomplete higher education	1	0.3	0.5	0.5	0.4	0.8	0.6	0.6
Complete higher education	6.2	8.9	5.4	6	4.8	7.1	4.8	5.2
No information	32.5	27	24.2	25.5	32.1	24.4	19.4	21.4
Smoking status								
Smoker	36.5	38	42.7	41.3	36.9	38.9	39.5	39.1
Ex-smoker	41.5	40.9	38.9	39.5	41.7	39.6	41.3	41.1
Never smoked	22	21.1	18.4	19.2	21.4	21.5	19.2	19.8
First treatment								
No information	17.1	2.8	4.2	5.3	8.9	3.1	4.2	4.4
None	47.4	8.6	21	21.7	64.4	8.7	25.2	25.9
Surgical	0	63.6	7.4	15.3	0	69.9	10	19.1
Non-surgical	35.6	24.9	67.4	57.8	26.7	18.3	60.5	50.6

Source: Hospital-based Cancer Registry (HCR), 2013 and 2019.

Table 4 shows the percentage distribution by sex, race/color, and education, which changed little between 2013 and 2019, and the mortality rate from LC in Brazil. There is a slight reduction in deaths in the younger age groups. For people aged 49 years or younger, the percentage goes from 8.4% in 2013 to 5.6% in 2019 and, among those aged 50 to 59 years old, from 17.9% to 14.6%. The mortality rate per 10 thousand inhabitants also varied little during the period, and despite showing a slight reduction between 2013 (1.50) and 2014 (1.46), it rose again, reaching 1.55 in 2019. In every year, a higher rate was noted for men (1.79 in 2019) than for women (1.32 in 2019).

**Table 4:** Percentage distribution of lung cancer mortality, according to Brazil’s socioeconomic characteristics. Mortality Information System (SIM), 2013-2019.

Characteristic	2013	2014	2015	2016	2017	2018	2019
						
	Distribution of deaths						
Total (n)	30,199	29,659	30,009	30,439	30,808	31,498	32,635
Sex							
Male	59.6	58.8	58.3	58.1	57.5	56.8	56.9
Female	40.4	41.2	41.7	41.9	42.5	43.2	43.1
Age group							
49 years or younger	8.4	7.7	6.9	6.7	6.2	5.8	5.6
50 to 59 years old	17.9	17.9	17.7	17.2	16.6	16.1	14.6
60 to 69 years old	27.1	28.1	29.1	29.1	30	30.3	30.6
70 to 79 years old	27.5	27.8	28.1	28.3	28.2	28.6	29
80 years or older	19.1	18.5	18.3	18.7	19	19.2	20.2
Race/color							
White	58	58.4	58.7	59.1	58.9	58.7	57.7
Black	5.7	5.7	5.9	5.7	6.1	6.4	6.7
Mixed	30.3	30.4	30.6	30.5	31	31	32.1
Yellow or Indigenous	1.2	0.9	0.9	0.9	0.9	0.9	0.9
Education							
No education	14.3	13.7	13	13.2	12.5	12.3	12.9
Incomplete primary	32.4	32.5	33.4	33.5	33.7	34.2	34.8
Complete primary or incomplete secondary	13	13.6	13.8	13.8	14.2	14	14.2
Complete secondary	11	11.5	12	12.3	12.7	13.8	14.1
Incomplete higher education	0.9	0.9	1.1	1	1.1	1.1	1.2
Complete higher education	5.6	5.9	5.9	6.5	6.7	7	7.1
Ignored	10.8	11.6	11.4	11.6	11.2	10.4	10.2
Mortality rate (10,000 inhabitants)							
Total	1.5	1.46	1.47	1.48	1.48	1.51	1.55
Sex							
Male	1.81	1.74	1.73	1.74	1.73	1.73	1.79
Female	1.2	1.19	1.21	1.22	1.25	1.28	1.32
Age group (years)							
49 or younger	0.16	0.14	0.13	0.13	0.12	0.11	0.11
50 to 59	2.66	2.54	2.47	2.38	2.27	2.21	2.04
60 to 69	6.54	6.38	6.4	6.22	6.25	6.21	6.25
70 to 79	12.71	12.21	12.06	11.87	11.48	11.44	11.5
80 or older	19.01	17.29	16.58	16.45	16.17	15.98	16.65
Race/color							
White	2.57	-	-	-	-	-	3.32
Black	1.31	-	-	-	-	-	1.27
Mixed	1.52	-	-	-	-	-	1.31
Yellow or Indigenous	1.82	-	-	-	-	-	1.38
Education (ESC2010)							
No education	3.63	-	-	-	-	-	3.36
Incomplete primary	2.23	-	-	-	-	-	2.49
Complete primary or incomplete secondary	1.77	-	-	-	-	-	2.08
Complete secondary	0.83	-	-	-	-	-	1.03
Incomplete higher education	0.41	-	-	-	-	-	0.44
Complete higher education	0.93	-	-	-	-	-	1

Source: Mortality Information System (SIM), preliminary population estimates prepared by the Ministry of Health/SVS/DAENT/CGIAE (2013 and 2019), and National Survey of Health (2013 and 2019).

Concerning age groups, mortality rates increased progressively with age, especially from the age of 60 onwards. Among those aged 49 years or younger in 2019, there was a rate of 0.11, which increased to 6.25 among those aged 60 to 69 years old and 16.65 among those aged 80 or older.

In 2019, there was a rate more than twice as high for White people (3.32) compared to Black people (1.27), mixed-race people (1.31), and Yellow or Indigenous people (1.38). Mortality progressively decreased with increasing education. In 2019, people with no education had a rate of 3.36, while for people with incomplete higher education, it reached 0.44, and for those who completed higher education, 1.00.

Table 5 summarizes the results of the data sources used. The proportion of smokers was noted to decrease proportionally with advancing age, and the mortality rate from LC increased inversely. A higher proportion of smokers was found in groups with less education, and similarly, the mortality rate was also higher in this population. Meanwhile, in the HCR, which includes individuals who have received a diagnosis, those aged 80 years or older represented 6.5%, and concerning deaths (SIM), this percentage was 20.2%.

**Table 5:** Percentage distribution by socioeconomic characteristics of the population (National Survey of Health [PNS]) and lung cancer cases (Hospital-based Cancer Registry [HCR]), according to smoking status, deaths, and mortality rate (Mortality Information System [SIM]). Brazil, 2019.

Characteristic	PNS (population)			HCR (LC)			Deaths	Mortality rate (10,000 inhabit.)
	
	Smokers	Never smoked	Population	Smokers	Never smoked	Total cases		
Total (n)	11,286	53,295	88,531	3,727	1,886	9,523	32,635	1.55
Total (% row)	12.7	60.2	100	39.1	19.8	100	–	
Sex								
Male	60.3	43.4	47.1	58.8	36.8	56.1	56.9	1.79
Female	39.7	56.6	52.9	41.2	63.2	43.9	43.1	1.32
Age group (years)								
49 or younger	53.6	64.6	56.6	5.6	15	6.9	5.6	0.11
50 to 59	22.7	15.3	17.7	24.8	17.3	19.5	14.6	2.04
60 to 69	16	10.9	14.2	36.8	27.2	34.7	30.6	6.25
70 to 79	6.1	6.4	8.1	29.4	32.5	32.5	29	11.5
80 or older	1.6	2.8	3.4	3.4	8	6.4	20.2	16.65
Race/color								
White	32.9	37.4	36.6	40.6	63.5	55.1	57.7	3.32
Black	13.4	10.7	11.4	5.7	4.6	5.6	6.7	1.27
Mixed	52.1	50.5	50.4	52.5	31.4	38.4	32.1	1.31
Yellow or Indigenous	1.6	1.4	1.5	1.2	0.4	0.2	0.9	1.38
Education								
No education	12.8	6.2	8.6	7.1	6.6	7	12.9	3.36
Incomplete primary	41.6	26.6	31.6	38.9	37	37.9	34.8	2.49
Complete primary or incomplete secondary	15.4	13.6	13.6	15.1	13.5	14.6	14.2	2.08
Complete secondary	18.5	30	26.4	12.8	15.7	13.3	14.1	1.03
Incomplete higher education	3.3	5.1	4.5	0.5	0.7	0.6	1.2	0.44
Complete higher education	8.3	18.5	15.4	4.8	6.2	5.2	7.1	1
Ignored	-	-	-	20.8	20.2	21.4	10.2	-

Source: National Survey of Health (PNS), 2019; Hospital-based Cancer Registry (HCR), 2019; Mortality Information System (SIM), 2019.

## DISCUSSION

In the general population, this study confirmed the worldwide downward trend in the prevalence of smokers^26^, as found in Brazilian capitals from VIGITEL^18^. Contrary to what was mentioned in that telephone survey, there was no continuity in reducing the prevalence of male smokers. Furthermore, the reduction only occurs in age groups up to 59 years old, among White and Yellow or Indigenous people. Regarding education, the most significant percentage reduction was observed among individuals with no education, with an increase among those with more education. It is essential to mention the reduction in the percentage of those who have never smoked, which may highlight the challenges in the sustainability of tobacco control policies^27^.

Regarding the smoking burden, between 2013 and 2019, a decrease was noted in the proportion of those who smoked more than 20 pack-years. In addition, women were noted to smoke less intensely than men, and individuals with less education have a more significant smoking burden. The international literature supports such findings^26,28^.

Behavioral patterns in tobacco consumption also varied by race/color. Black and mixed-race people have a lower smoking burden than White people. Despite this, they have a higher proportion of LC cases diagnosed at an advanced stage and, consequently, with a worse prognosis. This finding is similar to research carried out in the USA^9^. There are several hypotheses to explain this difference, including variation in metabolism^9^ and overlapping socioeconomic factors, such as a diet with insufficient amounts of fruits and vegetables^9^.

As expected, in the HCR, the percentage of individuals with LC who have never smoked is much lower than that found in the general population (PNS), proving that smoking is a critical risk factor for the development of LC^1^. Access to diagnosis and treatment of the disease is concentrated in the age groups between 50 and 80 years old. Regarding smoking among those with LC in the HCR, men are predominant. Women are the majority among those who have never smoked. A significant disparity in access to LC diagnosis and treatment was noted as, in the general population, mixed-race people predominantly with a history of smoking (PNS), and among those with LC registered at the HCR, the majority are White.

LC is still one of the leading preventable causes of death in the country and worldwide^1^. When analyzing deaths by SIM, the mortality rate from the disease is considerably higher for White people, in contrast to the evidence, which points to higher mortality rates among Black and mixed-race people as a result of inequality in access to timely diagnosis and treatment^29,30^.

Some factors may explain the discrepancy. The predominance of White people in the HCR likely highlights the difficulty in accessing treatment for Black and mixed-race people. In SIM, there may be errors in the registration of LC as the underlying cause of death^31^. Furthermore, another possible explanation could be the differences in the mortality profile between White and Black people, with the latter being more affected by external and infectious causes, leading to premature death^32^. It should also be noted that the registered race/color variable is mentioned in the various information systems, either by the individual or the health professional. Knowing that there is a collinearity between race/color and education^33^, these hypotheses are raised due to the inverse association identified in this research between education and the mortality rate due to LC since it progressively decreases with increasing years of education. Future studies should analyze this issue to elucidate this apparent discrepancy in Brazilian records.

Some corrections were made to analyze the SIM and HCR data. Despite the small effect, corrections are essential for better completeness of information in the SIM. The correction at the HCR for staging was fundamental, preventing a 32% missing.

This study uses secondary data sources and, therefore, is restricted to the variables existing in the databases and their quality. In the PNS, the calculation of smoking status and smoking burden is based on self-reported information, with possible memory bias, mainly concerning the age at which smoking started. Furthermore, there is also no measurement of the starting date of smoking for occasional smokers. The HCR covers hospital records and is not population-based. Additionally, even though it has smoking information, it does not include the smoking burden, making it impossible to know the smoking intensity for those diagnosed with LC. Another limitation is the differences in measuring race/color between the different sources used.

Notably, this is the first study using triangulation of national data sources for different time points. This allowed us to raise essential hypotheses about access to LC diagnosis and treatment in Brazil. Thus, this article made it possible to analyze the population distribution of smoking between 2013 and 2019. It also highlighted relevant sociodemographic disparities in access to diagnosis, treatment, and mortality due to LC.

Strengthening the Population-Based Cancer Registry (PBCR)^34^ is recommended to advance knowledge about LC in the country, expanding its coverage in Brazilian municipalities, as it enables linkage between data from various information systems and, thus, obtaining global, longitudinal, and accurate data, which leads to more specific analyses focusing on vulnerable populations to reduce disparities.

Furthermore, this work highlights the importance of feasibility studies on implementing an LC screening strategy in Brazil, as a high percentage of diagnosis was found at an advanced stage^2,4^. Studies indicate that combined prevention and early diagnosis strategies tend to work better in controlling mortality due to LC^35^.

Finally, it is highlighted that the approach to LC and its risk factors is multifaceted. It involves strengthening information systems to measure and reduce disparities, intervene for diagnosis and treatment at an early stage, and continually invest in tobacco control policies.
