# Supplementary material for: Smoking, mortality, access to diagnosis, and treatment of lung cancer in Brazil
Source: Rev Saude Publica. 2024 Apr 25;58:18. doi: 10.11606/s1518-8787.2024058005704 (PMC11090611; doi:10.11606/s1518-8787.2024058005704)
Supplement: Translation

# Translation: Tabagismo, mortalidade, acesso ao diagnóstico e tratamento de câncer de pulmão no Brasil

**Authors:** Mônica Rodrigues Campos, Jessica Muzy Rodrigues, Aline Pinto Marques, Lara Vinhal Faria, Tayná Sequeira Valerio, Mario Jorge Sobreira da Silva, Debora Castanheira Pires, Luisa Arueira Chaves, Carlos Henrique Dantas Cardoso, Silvio Rodrigues Campos, Isabel Cristina Martins Emmerick

**Affiliations:** I Brasil Fundação Oswaldo Cruz. Escola Nacional de Saúde Pública Sérgio Arouca. Departamento de Ciências Sociais. Rio de Janeiro, RJ, Brasil; II Brasil Fundação Oswaldo Cruz. Instituto de Comunicação e Informação Científica e Tecnológica em Saúde. Laboratório de Informação em Saúde. Rio de Janeiro, RJ, Brasil; III Brasil Fundação Oswaldo Cruz. Escola Nacional de Saúde Pública Sérgio Arouca. Programa de Pós-Graduação em Saúde Pública. Rio de Janeiro, RJ, Brasil; IV Brasil Instituto Nacional de Câncer. Divisão de Ensino. Rio de Janeiro, RJ, Brasil; V Brasil Fundação Oswaldo Cruz. Instituto Nacional de Infectologia Evandro Chagas. Laboratório de Pesquisa Clínica em DST e Aids. Rio de Janeiro, RJ, Brasil; VI Brasil Universidade Federal do Rio de Janeiro. Instituto de Ciências Farmacêuticas. Macaé, RJ, Brasil; VII Brasil Universidade Federal do Rio de Janeiro. Instituto de Educação em Ciências e Saúde. Programa de Pós-Graduação em Educação, Ciências e Saúde. Rio de Janeiro, RJ, Brasil; VIII Brasil Fundação Oswaldo Cruz. Escola Nacional de Saúde Pública Sérgio Arouca. Rio de Janeiro, RJ, Brasil; IX Estados Unidos University of Massachusetts. UMass Chan Medical School. Department of Surgery. Worcester, MA, Estados Unidos

**Keywords:** Tabagismo, Neoplasias Pulmonares, Sistemas de Informação em Saúde, Registros de Mortalidade

## Abstract

**INTRODUÇÃO:**

O câncer de pulmão (CP) é um relevante problema de saúde pública no Brasil e no mundo, dada sua alta incidência e mortalidade. Assim, objetiva-se analisar a distribuição do tabagismo e da carga tabágica segundo características sociodemográficas e disparidades no acesso, no tratamento e na mortalidade por CP no Brasil, em 2013 e 2019.

**MÉTODO:**

Estudo retrospectivo de triangulação de fontes de dados de abrangência nacional: a) análise da distribuição do tabagismo, baseada na Pesquisa Nacional de Saúde (PNS); b) investigação dos registros de CP, via Registros Hospitalares de Câncer (RHC); e c) distribuição da mortalidade por CP, no Sistema de Informação sobre Mortalidade (SIM).

**RESULTADOS:**

Verificou-se redução do percentual de pessoas que nunca fumaram de 2013 (68,5%) para 2019 (60,2%), assim como da carga tabágica (anos-maço). Esta foi observada maior em homens em pessoas de faixas etárias mais avançadas e de menor escolaridade. Em relação aos pacientes registrados no RHC, a entrada no serviço de saúde se dá a partir de 50 anos, e apenas 19% nunca fumaram. Ao passo que os fumantes na população são majoritariamente pardos, os pacientes no RHC são em maioria brancos. Quanto ao estadiamento inicial (I e II), é mais frequente em pessoas brancas e que nunca fumaram. A taxa de mortalidade apresentou variação de 1,00, para pessoas com ensino superior, a 3,36, entre pessoas sem instrução, assim como pessoas brancas têm uma taxa de mortalidade três vezes maior que a de pessoas negras e pardas.

**CONCLUSÃO:**

Este artigo apontou relevantes disparidades sociodemográficas no acesso ao diagnóstico, tratamento e mortalidade do CP. Assim, recomenda-se: fortalecer o Registro de Câncer de Base Populacional; desenvolver e implementar estratégia de *screening* de CP no Brasil, uma vez que a realização de estratégias de prevenção e diagnóstico precoce combinadas funcionam melhor no controle da mortalidade pela doença; e investimento contínuo nas políticas de prevenção e controle do tabagismo.

## INTRODUÇÃO

O câncer de pulmão (CP) é a doença maligna mais comum em todo o mundo desde 1985, tanto em incidência quanto em mortalidade^1^. Em 2020, foi estimado que cerca de 12% dos novos casos de câncer eram de pulmão e que ele foi responsável por 18,4% das mortes por câncer no mundo^1^. Além disso, observa-se alta letalidade e baixa sobrevida após o diagnóstico, especialmente em estágio avançado^2^.

No Brasil, as estimativas para o período 2023-2025 apontam que o CP será o segundo tipo de câncer mais comum, sem contar os cânceres específicos de mama e próstata^3^, e, em 2020, também foi responsável pela principal causa de mortalidade devido ao câncer^4^.

O principal fator de risco para o desenvolvimento de CP é o fumo^5^, frequentemente associado à idade, em razão do tempo de exposição ao tabaco^4^. Outros fatores relacionados são risco ocupacional^6^, exposição ambiental^7^ e características sociodemográficas, como sexo^8^, raça^9^ e escolaridade^10^. Ressalta-se que, no Brasil, há uma lacuna na literatura no que tange aos outros fatores que não o tabagismo.

Compreender como esses fatores afetam o risco de desenvolver CP, sua associação com o consumo de tabaco no Brasil e o possível impacto na mortalidade é essencial para a formulação adequada de políticas públicas. Uma maneira promissora de reduzir as disparidades do CP é melhorar a prevenção e a detecção da doença em estágio incial^11^, principalmente através de programas de rastreamento de pessoas em grupos de risco^12^. Os critérios para estabelecer um programa de rastreamento adequado dependem da compreensão acerca do grau de vulnerabilidade de uma população e das disparidades no seu acesso ao diagnóstico e tratamento^12^.

A idade e o tabagismo geralmente são os principais critérios para definição de grupos de alto risco nas diretrizes para rastreamento de CP. No entanto, esses parâmetros devem ser ajustados considerando a composição demográfica e suas especificidades, contemplando também outros marcadores relevantes como a situação socioeconômica^12^.

As diretrizes chinesas, por exemplo, foram adaptadas quanto à idade e carga tabágica (atualmente 50 a 74 anos, com 30 anos-maço), e considera os efeitos da poluição do ar pela exposição ambiental e fatores genéticos^13^. Nos EUA, as diretrizes da *United States Preventive Services Task Force* (USPSTF) foram atualizadas entre 2013 e 2021 para mitigar problemas de acesso de pessoas da raça/cor preta. A faixa etária foi ampliada para 50 a 80 anos (anteriormente 55 a 80 anos) e a carga de tabagismo foi reduzida de 30 para 20 anos-maço^14^.

Um valioso método para conhecer a população em risco é a triangulação por meio de bases secundárias, que além de facilmente acessíveis e com abrangência nacional, permitem tanto a caracterização sociodemográfica quanto a avaliação de aspectos de saúde e hábitos de vida. No Brasil, observa-se o uso sistemático e regular do Sistema de Informações sobre Mortalidade (SIM) para estudo do CP^4,15,16^. Entretanto, permanecem dois grandes desafios: estimar o risco do desenvolvimento do CP e mensurar suas disparidades no diagnóstico e tratamento.

Para a estimação do risco, o mais importante é o cálculo da carga tabágica, o que é possível através de dados da Pesquisa Nacional de Saúde (PNS)^17^. Apesar da Vigilância de Fatores de Risco e Proteção para Doenças Crônicas por Inquérito Telefônico (Vigitel) fornecer informações específicas sobre doenças crônicas e seus fatores de risco, sua aplicação é feita apenas em capitais, dificultando a extrapolação dos resultados a nível nacional^18^. Por sua vez, as disparidades no diagnóstico e tratamento podem ser mapeadas a partir dos Registros Hospitalares de Câncer (RHC), que possibilitam a análise segundo características sociodemográficas, epidemiológicas e clínicas^19^.

Com vistas a subsidiar o rastreamento adequado de CP no país, este artigo tem como objetivo analisar a distribuição populacional do tabagismo, a carga tabágica, os casos e mortalidade de CP e as disparidades em seu acesso e tratamento, segundo características sociodemográficas no Brasil, no período de 2013 a 2019.

## MÉTODOS

Foi realizado um estudo retrospectivo a partir de triangulação da PNS, RHC e SIM, como fontes de dados secundárias de abrangência nacional. O RHC é uma base nacional, de coleta sistemática e contínua de informações dos pacientes atendidos em unidades hospitalares com diagnóstico confirmado de câncer. O envio de dados é obrigatório para os hospitais habilitados na Atenção Especializada em Oncologia do Sistema Único de Saúde (SUS) e facultativo para os não habilitados^20^.

A triangulação de bases foi realizada seguindo as seguintes etapas:

- análise populacional da distribuição do tabagismo (fumantes, ex-fumantes, carga tabágica e nunca fumou) – PNS (2013 e 2019);
- investigação dos casos diagnosticados de CP segundo tabagismo e estadiamento (inicial e avançado) – RHC^20^ (2013 e 2019);
- distribuição dos óbitos e da taxa de mortalidade de CP – SIM (2013 a 2019).

Visando a melhora da qualidade dos dados utilizados, foram realizados: procedimentos de redistribuição dos *missings* de tabagismo e raça/cor por unidade federativa (UF) no RHC; correção dos dados ausentes na variável de estadiamento (RHC), por redistribuição, segundo sexo, faixa etária, tabagismo e primeiro tratamento; e, no SIM, correção dos códigos-lixo (*garbage code*) para CP, correção de causas mal definidas (CMD), correção de sub-registro e redistribuição dos *missings* por sexo, idade e UF. Os códigos-lixo da CID se referem a diagnósticos indefinidos ou incompletos que não apontam com precisão a causa de morte ou internação^21^.

Todas as análises foram descritas segundo variáveis demográficas e socioeconômicas: sexo, faixa etária, raça/cor e escolaridade. O sexo contemplou as categorias “masculino” ou “feminino” e a faixa etária classificada em: a) até 49 anos, b) 50 a 59 anos, c) 60 a 69 anos, d) 70 a 79 anos; e e) 80 anos ou mais. Para “raça/cor”, foram utilizadas apenas as categorias a) branca, b) preta ou c) parda, visto que o contingente de pessoas amarelas e indígenas na PNS foi muito pequeno, tanto em 2013 (1,6%) quanto em 2019 (2,5%). A variável escolaridade foi recategorizada em: a) sem instrução; b) fundamental incompleto; c) fundamental completo ou médio incompleto; d) médio completo, e) superior incompleto; e f) superior completo. Para calcular a taxa de mortalidade de CP por raça e escolaridade, foi necessário estimar a população brasileira para os anos de 2013 e 2019, aplicando-se a distribuição percentual das variáveis de escolaridade e raça/cor da PNS nas estimativas preliminares de população elaboradas pelo Ministério da Saúde/ Secretaria de Vigilância em Saúde (SVS)/ Departamento de Análise Epidemiológica e Vigilância de Doenças Não Transmissíveis (DASNT)/ Coordenação-Geral de Informações e Análises Epidemiológicas (CGIAE).

As análises para a PNS foram realizadas no *Statistical Package for Social Science for Windows* (SPSS) versão 21, para RHC no SAS *studio* e *phyton* e, para o SIM, utilizou-se SPSS versão 21 e *phyton*.

### Aspectos metodológicos da estimação do tabagismo e carga tabágica na PNS

Foram selecionados os moradores adultos (≥ 18 anos) que responderam ao questionário específico sobre seu estado de saúde, estilo de vida e doenças crônicas, o que totalizou 60.202 respondentes em 2013 e 88.943 em 2019. Utilizou-se a versão reponderada da PNS-2013, visando a comparação com 2019.

Quanto a definição/elaboração das variáveis advindas da PNS, para identificar os fumantes foi utilizada a pergunta: “Atualmente, o(a) Sr(a) fuma algum produto do tabaco?”. Foram considerados fumantes os que responderam “Sim, diariamente” ou “Sim, menos que diariamente”. Os ex-fumantes foram identificados a partir da pergunta: “E no passado, o(a) Sr(a) fumou algum produto do tabaco?”, considerando as respostas “Sim, diariamente” ou “Sim, menos que diariamente”. Os não fumantes foram os que responderam “Não, nunca fumei” a partir da pergunta anterior.

A carga tabágica foi estimada para pessoas que fumavam diariamente e que consumiam “somente cigarro industrializado”, “somente cigarro de palha ou enrolado” ou ambos. Os fumantes e ex-fumantes esporádicos não responderam às perguntas sobre idade de início do hábito de fumar e sobre a quantidade consumida. Aproximadamente 10% dos ex-fumantes (n = 2.102) reportaram parar de fumar antes da data de início do hábito de fumar, sendo desconsiderados no cálculo da carga tabágica.

A carga tabágica foi estimada em anos-maço. Esse número é uma medida sintética que combina a duração e a intensidade do tabagismo: a carga tabágica de um ano-maço corresponde ao fumo diário de 20 cigarros durante um ano^22^. Foi realizada conversão para equivalência entre cigarro industrializado e o cigarro de palha ou enrolado, cujo consumo foi multiplicado por três. Apesar da inexistência de literatura robusta sobre a equivalência entre eles, especialistas apontam que um cigarro de palha equivale a três industrializados^23,24^. O resultado classificado nas categorias: a) até 19 anos-maço, b) de 20 a 29 anos-maçoe c) 30 anos-maço ou mais.

### Aspectos metodológicos das correções do SIM

Realizou-se imputação dos dados ausentes (*missings*) de sexo e idade. Para imputação dos *missings* de sexo, utilizou-se a categoria de resposta mais frequente na base de dados (masculino – 810 casos). Para a idade, havia 4.719 casos não preenchidos dentre os 215.247 óbitos por CP entre maiores de 18 anos (2013 – 2019), que foram imputados utilizando a mediana das idades dos casos válidos.

Os códigos-lixo foram corrigidos, verificando-se os códigos de interesse para CP (CID C34) e sua porcentagem de redistribuição proposta por Malta e colaboradores^16^. Contabilizou-se a distribuição de códigos-lixo para aferir sua frequência absoluta em cada subgrupo, segundo macrorregião de residência e faixa etária, a serem utilizadas no destino da redistribuição. Após este levantamento, n = 4.719 óbitos tipo códigos-lixo foram redistribuídos.

Óbitos codificados como Causas Mal Definidas (CMD) se referem ao capítulo “R” do CID-10 (R00-R99). A redistribuição foi realizada de acordo com a distribuição proporcional de causas, verificada entre as CMD investigadas e reclassificadas, segundo o coeficiente proposto por França e colaboradores^15^, segundo região, faixa etária e sexo. Após a investigação para o CID-C34, n = 12.992 CMD foram redistribuídas.

A última etapa de correção no SIM foi a implementação do fator de correção dos sub-registro de óbitos, segundo metodologia proposta pela Rede Interagencial de Informações para a Saúde (RIPSA)^25^. O total quantificado depois da correção foi de n = 10.176 óbitos de CP em relação aos informados originalmente (n = 203.986).

Após a realização dos procedimentos mencionados, o percentual de correção no SIM foi de 0,03% na variável sexo, 2% para a idade, 2% nos códigos-lixo, 6% nas causas mal definidas e 4,7% nos sub-registros.

## RESULTADOS

A Tabela 1 apresenta a distribuição percentual de fumantes, ex-fumantes e pessoas que nunca fumaram e a carga tabágica, segundo variáveis sociodemográficas em 2013 e 2019. Verificou-se redução do percentual de pessoas que nunca fumaram de 2013 (68,5%) para 2019 (60,2%) e o aumento dos ex-fumantes de 17% (2013) para 27,1% (2019). O percentual de fumantes apresentou leve redução entre os anos, de 14,5% (2013) para 12,7% (2019).

**Tabela 1:** Distribuição percentual da população por características sociodemográficas, segundo tabagismo e carga tabágica na Pesquisa Nacional de Saúde (PNS). Brasil, 2013 e 2019.

Variáveis	Proporção de fumantes				Carga tabágica		
	
	Fumantes	Ex-fumantes	Nunca fumou	Total	Até 19 anos-maço	20 anos-maço ou mais	Total
2013							
Total (n)	8.729	10.258	41.215	60.202	9.699	5.324	15.023
Total (% linha)	14,5	17	68,5	100	64,6	35,4	100
Sexo							
Homem	57,2	52,6	37,7	43,1	50,5	63,7	55,2
Mulher	42,8	47,4	62,3	56,9	49,5	36,3	44,8
Faixa etária							
49 anos ou menos	62,1	44,6	72,8	66,4	58,5	32,9	49,4
50 a 59 anos	21,8	22,4	11,7	15	19,2	31,1	23,4
60 a 69 anos	10,9	18,6	8,2	10,4	12,8	22,3	16,1
70 a 79 anos	4,2	10,5	4,8	5,7	6,8	10,8	8,2
80 anos ou mais	1,1	3,9	2,4	2,5	2,7	3	2,8
Cor ou raça							
Branca	35,5	39,9	41	40	37,7	41,1	38,9
Preta	11,4	9,1	9	9,4	10,3	10,2	10,3
Parda	51,3	49,2	48,5	49	50,2	47,3	49,2
Amarela ou indígena	1,7	1,8	1,5	1,6	1,8	1,4	1,7
Escolaridade							
Sem instrução	14,2	14,7	7,4	9,6	13,5	18,4	15,2
Fundamental incompleto	41,1	39,8	25,7	30,4	39	46,3	41,6
Fundamental completo ou médio incompleto	16	13,3	15,7	15,3	15,9	11,6	14,4
Médio completo	17,9	18,8	30,6	26,8	18,9	14	17,1
Superior incompleto	3,4	3,1	5,9	5	3,5	2	2,9
Superior completo	7,5	21,6	21,2	20,5	9,2	7,7	8,7
2019							
Total (n)	11.286	23.950	53.295	88.531	21.587	9.362	30.949
Total (% linha)	12,7	27,1	60,2	100	69,8	30,2	100
Sexo							
Homem	60,3	48,8	43,4	47,1	46,9	63,5	51,9
Mulher	39,7	51,2	56,6	52,9	53,1	36,5	48,1
Faixa etária (anos)							
49 ou menos	53,6	40,4	64,6	56,6	51,6	23,1	42,9
50 a 59	22,7	20,7	15,3	17,7	19,3	27,6	21,8
60 a 69	16	20,6	10,9	14,2	15,5	29,3	19,7
70 a 79	6,1	12,8	6,4	8,1	9,3	15,1	11,1
80 ou mais	1,6	5,5	2,8	3,4	4,3	4,9	4,5
Cor ou raça							
Branca	32,9	36,6	37,4	36,6	34,4	39,1	35,8
Preta	13,4	12,2	10,7	11,4	12,9	12,3	12,7
Parda	52,1	49,5	50,5	50,4	51,1	47,2	49,9
Amarela ou indígena	1,6	1,7	1,4	1,5	1,7	1,4	1,6
Escolaridade							
Sem instrução	12,8	12	6,2	8,6	11,3	15,6	12,6
Fundamental incompleto	41,6	37,8	26,6	31,6	36,5	47,5	39,8
Fundamental completo ou médio incompleto	15,4	12,7	13,6	13,6	14,2	11,5	13,4
Médio completo	18,5	22,2	30	26,4	22,9	15,3	20,6
Superior incompleto	3,3	3,5	5,1	4,5	4	1,7	3,3
Superior completo	8,3	11,8	18,5	15,4	11	8,5	10,2

Fonte: Pesquisa Nacional de Saúde (PNS), 2013 e 2019.

Há redução da carga tabágica entre 2013 e 2019, e mulheres consistentemente fumam menos que homens. Intensificou-se a participação masculina entre os fumantes, que já era alta em 2013 (57,2%), com expressiva carga tabágica acima de 20 anos-maço(63,7%). A maior parcela dos que “nunca fumaram” era feminina em 2013 (62,3%) e 2019 (56,6%). A distribuição dos ex-fumantes por sexo é similar em ambos os anos.

Foi identificado crescimento do percentual de ex-fumantes a partir dos 60 anos entre 2013-2019. Em contrapartida, em 2019, a carga tabágica entre pessoas dessa faixa etária foi mais alta. A distribuição interna de fumantes e da carga tabágica segundo raça/cor é similar à distribuição geral da população em ambos os anos. Considerando-se a raça/cor, indivíduos negros ou pardos tem menor carga tabágica quando comparados aos brancos, especialmente para o ano de 2019. Destaca-se também a alta carga tabágica em pessoas de menor escolaridade em ambos os anos.

A Tabela 2 apresenta a distribuição percentual de fumantes, ex-fumantes e pessoas que nunca fumaram entre os pacientes registrados no RHC, em 2013 e 2019. No RHC, o percentual de pessoas que nunca fumaram era, em média, 19%, o que é consideravelmente inferior ao observado na população geral (68,5% em 2013 e 60,2% em 2019). A entrada no serviço de saúde se dá a partir de 50 anos, e o percentual de pessoas na faixa etária mais avançada (80 anos ou mais) é extremamente baixo em relação às demais idades, especialmente entre fumantes (3,4% nos dois anos).

**Tabela 2:** Distribuição percentual dos pacientes diagnosticados com câncer de pulmão no Registro Hospitalar de Câncer (RHC) por características sociodemográficas, segundo tabagismo. Brasil, 2013 e 2019.

Variáveis	2013				2019			
	
	Fumantes	Ex-fumantes	Nunca fumou	Total	Fumantes	Ex-fumantes	Nunca fumou	Total
Total (n)	4.623	4.413	2.147	11.183	3.727	3.910	1.886	9.523
Total (% linha)	41,3	39,5	19,2	100	39,1	41,1	19,8	100
Sexo								
Homem	62	65,2	38,7	58,8	58,8	65,4	36,8	57,1
Mulher	38	34,2	61,3	41,2	41,2	34,6	63,2	42,9
Faixa etária (anos)								
49 ou menos	7,8	5,3	20,2	9,2	5,6	4,1	15	6,9
50 a 59	29,2	20,5	20	24	24,8	16,9	17,3	20,1
60 a 69	31,6	32,6	24,3	30,5	36,8	36,7	27,2	34,9
70 a 79	28,1	34,5	27,4	30,5	29,4	34,5	32,5	32,1
80 ou mais	3,4	7,1	8,1	5,8	3,4	7,9	8	6,1
Cor ou raça								
Branca	41,7	66,2	68,7	56,5	40,6	65,6	63,5	55,3
Preta	6,1	6,5	4,4	5,9	5,7	6,3	4,6	5,7
Parda	51,1	26,5	26,5	36,7	52,5	27,2	31,4	38
Amarela ou indígena	1,1	0,8	0,4	0,9	1,2	0,9	0,4	0,9
Escolaridade								
Sem instrução	7,8	8,8	7,8	8,2	7,1	7,1	6,6	7
Fundamental incompleto	33,3	33,4	31,1	32,9	38,9	37,4	37	37,9
Fundamental completo ou médio incompleto	15,2	15,7	16,6	15,6	15,1	14,6	13,5	14,6
Médio completo	10,6	10,8	13,5	11,3	12,8	12,7	15,7	13,3
Superior incompleto	0,4	0,5	0,8	0,5	0,5	0,7	0,7	0,6
Superior completo	6,2	5,8	5,9	6	4,8	5,1	6,2	5,2
Sem informação	26,5	9,8	4,7	25,5	20,8	22,4	20,2	21,4

Fonte: Registros Hospitalares de Câncer (RHC), 2013 e 2019.

Assim como na PNS, na distribuição por sexo dos fumantes e ex-fumantes há um predomínio dos homens, tanto em 2013 (62% e 65,2%) quanto em 2019 (58,8% e 65,4%). Entre os que nunca fumaram, as mulheres são maioria (61,3% em 2013 e 63,2% em 2019). Destaca-se a diferença entre a distribuição por raça/cor, com predomínio de pardos na PNS (49% em 2013 e 50,4% em 2019) e majoritariamente brancos entre os pacientes no RHC (56,5% em 2013 e 55,3% em 2019).

Apesar do alto percentual de pardos fumantes e ex-fumantes na população geral (Tabela 1), eles não são captados da mesma maneira no RHC, apontando uma sub-representação. Em contrapartida, os brancos, mesmo ex-fumantes ou que nunca fumaram, tem mais acesso ao diagnóstico e tratamento, representando uma parcela maior no RHC em relação à distribuição populacional (PNS). Quanto à escolaridade, chama atenção o alto percentual de incompletude no preenchimento dessa variável nos anos (25,5% e 21,4%), em detrimento da sua importância como *proxy* socioeconômica para análise das disparidades de acesso.

A Tabela 3 apresenta o grau de estadiamento do CP nos pacientes do RHC em 2013 e 2019. Destaca-se o baixo percentual de pessoas com grau estadiamento inicial (I e II) em 2013 (14,2%) e 2019 (14,5). A distribuição pelas características sociodemográficas da população que acessa os serviços é similar entre os anos analisados. Em 2019, o percentual de pessoas brancas, com ensino superior e que nunca fumaram foi superior nos graus de estadiamento I e II (58,2%, 6,8% e 22,6%). Em relação ao tratamento, observa-se elevado percentual de primeiro tratamento cirúrgico em pessoas com estadiamento I e II em 2013 (45,6%) e 2019 (50,2%).

**Tabela 3:** Distribuição percentual dos pacientes diagnosticados com câncer de pulmão no Registro Hospitalar de Câncer (RHC) por características sociodemográficas, segundo grau de estadiamento inicial (I e II) e avançado (III e IV). Brasil, 2013 e 2019.

Variáveis	2013				2019			
	
	Sem informação	I e II	III e IV	Total	Sem informação	I e II	III e IV	Total
Total (n)	1.094	1.705	8.384	11.183	832	1.587	7.104	9.523
Total (% linha)	9,8	15,2	75	100	8,7	16,7	74,6	100
Sexo								
Homem	55,8	55,8	59,8	58,8	57,7	50,7	57,1	56,1
Mulher	44,2	44,2	40,2	41,2	42,3	49,3	42,9	43,9
Faixa etária (anos)								
49 ou menos	10,7	9,7	8,9	9,2	7,7	6,9	6,7	6,9
50 a 59	21,8	21,6	24,8	24	17,7	17,4	20,2	19,5
60 a 69	27,4	31,3	30,8	30,6	34	34,7	34,8	34,7
70 a 79	32,2	32,4	29,9	30,5	30,8	34,4	32,3	32,5
80 ou mais	8	5	5,6	5,8	9,9	6,7	6	6,4
Cor ou raça								
Branca	56,8	57,9	56,2	56,5	54,1	57,9	54,6	55,1
Preta	4,9	6,5	5,9	5,9	5,9	4,1	5,9	5,6
Parda	37,3	34,6	37	36,7	38,8	36,9	38,7	38,4
Amarela ou indígena	1	1,1	0,9	0,9	1,2	1,1	0,8	0,2
Escolaridade								
Sem instrução	9,7	7,6	8,1	8,2	8,2	5,6	7,2	7
Fundamental incompleto	27,5	30,1	34,2	32,9	33,1	33,3	39,5	37,9
Fundamental completo ou médio incompleto	14,2	14,4	16,1	15,6	3	12	45	14,6
Médio completo	9	11,7	11,5	11,3	9,1	14,9	15,1	13,3
Superior incompleto	1	0,3	0,5	0,5	0,4	0,8	0,6	0,6
Superior completo	6,2	8,9	5,4	6	4,8	7,1	4,8	5,2
Sem informação	32,5	27	24,2	25,5	32,1	24,4	19,4	21,4
Tabagismo								
Fumante	36,5	38	42,7	41,3	36,9	38,9	39,5	39,1
Ex-fumante	41,5	40,9	38,9	39,5	41,7	39,6	41,3	41,1
Nunca fumou	22	21,1	18,4	19,2	21,4	21,5	19,2	19,8
Primeiro tratamento								
Sem informação	17,1	2,8	4,2	5,3	8,9	3,1	4,2	4,4
Nenhum	47,4	8,6	21	21,7	64,4	8,7	25,2	25,9
Cirúrgico	0	63,6	7,4	15,3	0	69,9	10	19,1
Não cirúrgico	35,6	24,9	67,4	57,8	26,7	18,3	60,5	50,6

Fonte: Registros Hospitalares de Câncer (RHC), 2013 e 2019.

A Tabela 4 apresenta a distribuição percentual por sexo, raça/cor e escolaridade, que pouco mudou entre 2013 e 2019, e a taxa de mortalidade por CP no Brasil. e. Nota-se leve redução dos óbitos nas faixas etárias mais jovens. Para pessoas com 49 anos ou menos, o percentual passa de 8,4% em 2013 para 5,6% em 2019 e, entre os de 50 a 59 anos, passa de 17,9% para 14,6%. A taxa de mortalidade por 10 mil habitantes também variou pouco no período e, apesar de apresentar leve redução entre 2013 (1,50) e 2014 (1,46), volta a subir, chegando a 1,55 em 2019. Verificou-se em todos os anos uma taxa mais elevada para os homens (1,79 em 2019) em relação às mulheres (1,32 em 2019).

**Tabela 4:** Distribuição percentual da mortalidade por câncer de pulmão, segundo características socioeconômicas no Brasil. Sistema de Informação sobre Mortalidade (SIM), 2013-2019.

Variáveis	2013	2014	2015	2016	2017	2018	2019
						
	Distribuição dos óbitos						
Total (n)	30.199	29.659	30.009	30.439	30.808	31.498	32.635
Sexo							
Masculino	59,6	58,8	58,3	58,1	57,5	56,8	56,9
Feminino	40,4	41,2	41,7	41,9	42,5	43,2	43,1
Faixa etária							
49 anos ou mesmo	8,4	7,7	6,9	6,7	6,2	5,8	5,6
50 a 59 anos	17,9	17,9	17,7	17,2	16,6	16,1	14,6
60 a 69 anos	27,1	28,1	29,1	29,1	30	30,3	30,6
70 a 79 anos	27,5	27,8	28,1	28,3	28,2	28,6	29
80 anos ou mais	19,1	18,5	18,3	18,7	19	19,2	20,2
Raça/cor							
Branca	58	58,4	58,7	59,1	58,9	58,7	57,7
Preta	5,7	5,7	5,9	5,7	6,1	6,4	6,7
Parda	30,3	30,4	30,6	30,5	31	31	32,1
Amarela ou indígena	1,2	0,9	0,9	0,9	0,9	0,9	0,9
Escolaridade							
Sem instrução	14,3	13,7	13	13,2	12,5	12,3	12,9
Fundamental incompleto	32,4	32,5	33,4	33,5	33,7	34,2	34,8
Fundamental completo ou médio incompleto	13	13,6	13,8	13,8	14,2	14	14,2
Médio completo	11	11,5	12	12,3	12,7	13,8	14,1
Superior incompleto	0,9	0,9	1,1	1	1,1	1,1	1,2
Superior completo	5,6	5,9	5,9	6,5	6,7	7	7,1
Ignorado	10,8	11,6	11,4	11,6	11,2	10,4	10,2
Taxa de mortalidade (10.000 habitantes)							
Total	1,5	1,46	1,47	1,48	1,48	1,51	1,55
Sexo							
Masculino	1,81	1,74	1,73	1,74	1,73	1,73	1,79
Feminino	1,2	1,19	1,21	1,22	1,25	1,28	1,32
Faixa etária (anos)							
49 ou mesmo	0,16	0,14	0,13	0,13	0,12	0,11	0,11
50 a 59	2,66	2,54	2,47	2,38	2,27	2,21	2,04
60 a 69	6,54	6,38	6,4	6,22	6,25	6,21	6,25
70 a 79	12,71	12,21	12,06	11,87	11,48	11,44	11,5
80 ou mais	19,01	17,29	16,58	16,45	16,17	15,98	16,65
Raça/cor							
Branca	2,57	-	-	-	-	-	3,32
Preta	1,31	-	-	-	-	-	1,27
Parda	1,52	-	-	-	-	-	1,31
Amarela ou indígena	1,82	-	-	-	-	-	1,38
Escolaridade (ESC2010)							
Sem instrução	3,63	-	-	-	-	-	3,36
Fundamental incompleto	2,23	-	-	-	-	-	2,49
Fundamental completo ou médio incompleto	1,77	-	-	-	-	-	2,08
Médio completo	0,83	-	-	-	-	-	1,03
Superior incompleto	0,41	-	-	-	-	-	0,44
Superior completo	0,93	-	-	-	-	-	1

Fonte: Sistema de Informações sobre Mortalidade (SIM), estimativas preliminares de população elaboradas pelo Ministério da Saúde/SVS/DASNT/CGIAE (2013 e 2019) e Pesquisa Nacional de Saúde (2013 e 2019).

Em relação à faixa etária, as taxas de mortalidade aumentam progressivamente com a idade, especialmente a partir dos 60 anos. Entre aqueles com 49 anos ou menos, verificou-se, em 2019, uma taxa de 0,11, que aumenta para 6,25 entre os de 60 a 69 anos e 16,65 entre os de 80 anos ou mais.

Em 2019, observou-se uma taxa mais de duas vezes superior para as pessoas brancas (3,32) em relação aos pretos (1,27), pardos (1,31) e amarelos ou indígenas (1,38). A mortalidade diminui progressivamente com o aumento da escolaridade. Em 2019, as pessoas sem instrução apresentavam taxa de 3,36, enquanto para as pessoas com ensino superior incompleto chega a 0,44 e superior completo, 1,00.

A Tabela 5 sumariza os resultados das fontes de dados utilizadas. Pode-se observar que a proporção de fumantes diminui proporcionalmente ao avanço da idade e a taxa de mortalidade por CP aumenta inversamente. Assim como se encontrou maior proporção de fumantes nos grupos com menos escolaridade, a taxa de mortalidade também é maior nessa população. Enquanto isso, no RHC, que inclui os indivíduos que receberam diagnóstico, aqueles com 80 anos ou mais representam 6,5% e, em relação aos óbitos (SIM), esse percentual é de 20,2%.

**Tabela 5:** Distribuição percentual por características socioeconômicas da população (Pesquisa Nacional de Saúde [PNS]) e dos casos de câncer de pulmão (Registro Hospitalar do Câncer [RHC]), segundo tabagismo, dos óbitos e da taxa de mortalidade (Sistema de Informações sobre Mortalidade [SIM]). Brasil, 2019.

Variáveis	PNS (população)			RHC (CP)			Óbitos	Taxa mortalidade (10 mil hab.)
	
	Fumantes	Nunca fumou	População	Fumantes	Nunca fumou	Total de casos		
Total (n)	11.286	53.295	88.531	3.727	1.886	9.523	32.635	1,55
Total (% linha)	12,7	60,2	100	39,1	19,8	100	–	
Sexo								
Masculino	60,3	43,4	47,1	58,8	36,8	56,1	56,9	1,79
Feminino	39,7	56,6	52,9	41,2	63,2	43,9	43,1	1,32
Faixa etária (anos)								
49 ou mesmo	53,6	64,6	56,6	5,6	15	6,9	5,6	0,11
50 a 59	22,7	15,3	17,7	24,8	17,3	19,5	14,6	2,04
60 a 69	16	10,9	14,2	36,8	27,2	34,7	30,6	6,25
70 a 79	6,1	6,4	8,1	29,4	32,5	32,5	29	11,5
80 ou mais	1,6	2,8	3,4	3,4	8	6,4	20,2	16,65
Raça/cor								
Branca	32,9	37,4	36,6	40,6	63,5	55,1	57,7	3,32
Preta	13,4	10,7	11,4	5,7	4,6	5,6	6,7	1,27
Parda	52,1	50,5	50,4	52,5	31,4	38,4	32,1	1,31
Amarela ou indígena	1,6	1,4	1,5	1,2	0,4	0,2	0,9	1,38
Escolaridade								
Sem instrução	12,8	6,2	8,6	7,1	6,6	7	12,9	3,36
Fundamental incompleto	41,6	26,6	31,6	38,9	37	37,9	34,8	2,49
Fundamental completo ou médio incompleto	15,4	13,6	13,6	15,1	13,5	14,6	14,2	2,08
Médio completo	18,5	30	26,4	12,8	15,7	13,3	14,1	1,03
Superior incompleto	3,3	5,1	4,5	0,5	0,7	0,6	1,2	0,44
Superior completo	8,3	18,5	15,4	4,8	6,2	5,2	7,1	1
Ignorado	-	-	-	20,8	20,2	21,4	10,2	-

Fonte: Pesquisa Nacional de Saúde (PNS), 2019; Registros Hospitalares de Câncer (RHC), 2019; Sistema de Informações sobre Mortalidade (SIM), 2019.

## DISCUSSÃO

Na população geral, este estudo confirmou a tendência mundial decrescente da prevalência de fumantes^26^, conforme o encontrado nas capitais brasileiras a partir do Vigitel^18^. Ao contrário do citado neste inquérito telefônico, não se observou continuidade na redução da prevalência de homens fumantes. Além disso, a redução se dá apenas nas faixas etárias até 59 anos, entre pessoas brancas e amarelas ou indígenas. No tocante à escolaridade, a maior redução percentual foi observada entre indivíduos sem instrução, com aumento entre os mais escolarizados. É importante mencionar a redução no percentual dos que nunca fumaram, o que pode evidenciar os desafios na sustentabilidade das políticas de controle do tabagismo^27^.

Quanto à carga tabágica, notou-se que, entre os anos de 2013 e 2019, houve diminuição da proporção dos que fumam mais de 20 anos-maço. Observou-se, ainda, que mulheres fumam com menor intensidade que homens e que indivíduos com menor escolaridade têm maior carga tabágica, achados corroborados na literatura internacional^26,28^.

Os padrões de comportamento no consumo de tabaco também variaram por raça/cor. Pessoas pretas e pardas têm carga tabágica inferior à de pessoas brancas. Apesar disso, apresentam maior proporção de casos de CP diagnosticados em estágio avançado, e consequentemente, com pior prognóstico. Este achado é similar ao encontrado em pesquisas realizadas nos EUA^9^. Existem diversas hipóteses para explicar esta diferença, entre elas variação no metabolismo^9^ e superposição de fatores socioeconômicos, como uma dieta com quantidades insuficientes de frutas e legumes^9^.

Assim como esperado, no RHC o percentual de indivíduos com CP que nunca fumaram é muito inferior ao encontrado na população em geral (PNS), comprovando que o tabagismo é um fator de risco importantíssimo para o desenvolvimento do CP^1^. O acesso ao diagnóstico e tratamento da doença se concentra nas faixas etárias entre 50 e 80 anos. Em relação ao tabagismo dentre aqueles com CP no RHC, há um predomínio de homens, as mulheres são maioria entre aqueles que nunca fumaram. Notou-se uma importante disparidade no acesso ao diagnóstico e tratamento do CP pois, na população geral, há um predomínio de pardos com história de fumo (PNS), e entre aqueles com CP registrados no RHC, a maioria é composta por brancos.

O CP ainda é uma das principais causas de morte evitáveis no país e no mundo^1^. Quando analisados os óbitos pelo SIM, a taxa de mortalidade pela doença é consideravelmente maior para os brancos, em contraste com as evidências, que apontam maiores taxas de mortalidade entre pessoas pretas e pardas, em consequência da desigualdade no acesso ao diagnóstico e tratamento em tempo adequado ^29,30^.

Alguns fatores podem explicar a discrepância. É provável que a predominância de pessoas brancas no RHC evidencie a dificuldade no acesso ao tratamento de pessoas pretas e pardas. No SIM, é possível que haja falhas no registro de CP como causa básica de óbito^31^. Além disso, outra possível explicação pode ser as diferenças no perfil de mortalidade entre pessoas brancas e pretas, sendo as últimas mais atingidas por causas externas e infectocontagiosas, levando a morte prematura^32^. Ressalta-se, ainda, que nos vários sistemas de informação a variável raça/cor registrada é referida, seja pelo próprio indivíduo ou pelo profissional de saúde. Sabendo que há uma colinearidade entre raça/cor e escolaridade^33^, essas hipóteses são levantadas em função da associação inversa, identificada nesta pesquisa, entre escolaridade e a taxa de mortalidade por CP, uma vez que esta diminui progressivamente com o aumento dos anos de estudo. Estudos futuros devem analisar essa questão, para elucidar essa aparente discrepância nos registros brasileiros.

A fim de analisar os dados do SIM e do RHC, foram realizadas algumas correções. No SIM, apesar do diminuto efeito, as correções são importantes para melhor completude da informação. Já a correção realizada no RHC para o estadiamento foi fundamental, evitando 32% de perda.

Este estudo utiliza fontes de dados secundárias e, por conseguinte, está restrito às variáveis existentes nas bases e a qualidade delas. Na PNS, o cálculo do tabagismo e carga tabágica partem de informação autorreferida, com possível viés de memória, principalmente em relação a idade de início de fumo, e também não há aferição do início da data de fumo para fumantes eventuais. O RHC possui cobertura dos registros dos hospitais, não sendo de base populacional. Adicionalmente, ainda que possua a informação de tabagismo, não inclui a carga tabágica, impossibilitando conhecer a intensidade de fumo para os diagnosticados com CP. Outra limitação se refere às diferenças na aferição da raça/cor entre as diferentes fontes utilizadas.

Ressalta-se que este é o primeiro estudo utilizando triangulação de fontes de dados de abrangência nacional para diferentes momentos no tempo, o que permitiu levantar importantes hipóteses sobre o acesso ao diagnóstico e tratamento de CP no Brasil. Assim, este artigo possibilitou a análise da distribuição populacional do tabagismo na comparação dos anos de 2013 e 2019, além de apontar relevantes disparidades sociodemográficas no acesso ao diagnóstico, tratamento e mortalidade por CP.

Para que se avance no conhecimento do CP no país, recomenda-se fortalecer o Registro de Câncer de Base Populacional (RCBP)^34^, ampliando sua cobertura nos municípios brasileiros, pois possibilita a ligação (*linkage*) entre os dados de vários sistemas de informação e, assim, a obtenção de dados globais, longitudinais e acurados, que levam a análises mais específicas e com foco em populações vulneráveis, para reduzir as disparidades.

Além disso, este trabalho aponta a importância de estudos de viabilidade sobre a implementação de estratégia de rastreamento de CP no Brasil, uma vez que foi encontrado elevado percentual de diagnóstico em estágio avançado^2,4^. De fato, pesquisas apontam que a realização de estratégias de prevenção e diagnóstico precoce combinadas tendem a funcionar melhor no controle da mortalidade por CP^35^.

Finalmente, destaca-se que a abordagem do CP e seus fatores de risco é multifacetada, envolvendo o fortalecimento dos sistemas de informação para: mensurar e reduzir as disparidades; intervir para o diagnóstico e tratamento em estágio inicial; e investir continuamente nas políticas de controle do tabagismo.
